# Assessment of Crystals in the Synovial Fluid of Psoriatic Arthritis Patients in Relation to Disease Activity

**DOI:** 10.3390/diagnostics12051260

**Published:** 2022-05-18

**Authors:** Mariela Geneva-Popova, Stanislava Popova-Belova, Velicka Popova, Nikolay Stoilov

**Affiliations:** 1 Department of Propedeutic of Internal Diseases, Faculty of Medicine, Medical University of Plovdiv, Clinic of Rheumatology, University General Hospital “Sveti Georgi”, 4001 Plovdiv, Bulgaria; stanislava.popova@mu-plovdiv.bg; 2 Department of Propedeutic of Internal Diseases, Faculty of Medicine, Medical University of Plovdiv, University General Hospital “Kaspela”, 4001 Plovdiv, Bulgaria; drvpopova@gmail.com; 3 Department of Rheumatology, Faculty of Medicine, Medical University of Sofia, University General Hospital “St. Ivan Rilski”, 1431 Sofia, Bulgaria; dr_nstoilov@yahoo.com

**Keywords:** biomarkers, disease activity indices, psoriatic arthritis, synovial fluid crystals

## Abstract

Background: This study examines the relationship between the presence of crystals in the synovial fluid of patients with psoriatic arthritis (PsA) and disease activity. Methods: The synovial fluid of 156 PsA patients was analyzed and compared to 50 patients with gonarthrosis (GoA). The Leica DM4500P polarization microscope was used for crystal detection. Results: The presence of crystals was observed in 23.71% of PsA patients and none of the GoA patients, *p* < 0.001. Monosodium urate crystals (67.58%) and calcium pyrophosphate crystals (21.62%) were prevalent. The presence of crystals in the synovial fluid of PsA patients was associated with high disease activity according to the Composite Psoriatic Disease Activity Index (OR = 18.75, 95%; CI: 7.13 to 49.25) and the Disease Activity for Psoriatic Arthritis (OR = 15.96, 95%; CI: 5.76 to 44.23), with severe disability according to the Health Assessment Questionnaire Disability Index (OR = 13.60, 95%; CI: 5.09 to 36.31), and with severe pain on the Visual Analog Scale (OR = 157.25, 95%; CI: 39.50 to 625.94). Conclusion: Our results suggest that synovial fluid examination should be included in the treatment pathway for PsA patients with active disease, to aid in determining whether urate-lowering therapy is required.

## 1. Introduction

Psoriatic arthritis (PsA) is a chronic inflammatory joint disease that can seriously impact patients’ health-related quality of life [1]. Comorbidities associated with PsA include cardiovascular disease, metabolic syndrome, diabetes, and hyperuricemia [2,3,4]. The latter has been discussed in a number of studies that found elevated uric acid levels in the serum of PsA patients [5,6,7]. Furthermore, hyperuricemia has been linked to increased inflammation and disease severity in PsA patients [5,6]. According to Tsuruta et al., (2017), hyperuricemia is an independent risk factor for the development of PsA, which increases the crystallization of uric acid in and around the joints and leads to arthritis [8].

Synovial fluid (SF) analysis has shown good diagnostic potential for detecting crystal arthropathy; however, it is less commonly used in clinical practice and related research [9]. In rheumatology publications, information on the characteristics of the synovial fluid of PsA patients is relatively scarce [10,11].

Most of the research about hyperuricemia in PsA is based on evaluations of serum uric acid (SUA) levels [12,13,14]. Furthermore, Tripolino et al., observed in a review article that the findings of these studies are frequently contradictory, as some showed an association between hyperuricemia and disease state in PsA while others found no association [5]. Research involving the assessment of synovial fluid in PsA patients can shed more light on this inconsistency and aid in the development of more effective methods of diagnosis and treatment.

The aim of this study is to examine two main issues: (1) the prevalence of synovial fluid crystals in PsA patients versus a control group of gonarthrosis (GoA) patients, and (2) the relationship between the presence of crystals in the synovial fluid of PsA patients and disease activity indices including: the Disease Activity for Psoriatic Arthritis (DAPSA), the Psoriatic Composite Psoriatic Disease Activity Index (PASDAS), the Composite Psoriatic Disease Activity Index (mCPDAI), and the Health Assessment Questionnaire Disability Index (HAQ-DI).

## 2. Materials and Methods

This was a retrospective case–control study, involving 156 patients with a PsA diagnosis according to the CASPAR criteria and 50 GoA patients. All participants were diagnosed and treated in the Departments of Rheumatology at the University General Hospital “St. George” and the University General Hospital “Kaspela” in Plovdiv, Bulgaria. Data collection took place between July and December 2021. Only PsA patients with peripheral polyarticular asymmetrical joint involvement were included because they constituted the majority of those who visited the hospital during the recruitment period. Due to the small number of patients with other PsA subtypes, it was not possible to form representative subgroups. The inclusion criteria for the PsA patients were: (1) proven psoriatic arthritis with synovial effusion; (2) psoriatic arthritis treated with a biological agent (TNF-α-blocker); (3) absence of mental health comorbidities; (4) signed informed consent form for participation in the study. The restriction described in (2) above was imposed due to the small number of PsA patients treated with other therapies, which prevented the formation of treatment subgroups.

The following exclusion criteria were applied: (1) refusal to give informed consent; (2) diagnosis of a rheumatic disease other than PsA; (3) psoriatic arthritis treated with biologics other than adalimumab or etanercept; (4) decompensated cardiovascular, pulmonary or renal failure; (5) pregnant or lactating women.

The GoA patients were included if they met the following criteria (ACR, 1991): (1) knee pain of at least 5 years, over 50 years of age, stiffness of less than 30 min, crepitations in the knee, deformity and enlargement of the joint, without warming; (2) radiographic evidence of osteophytosis of the knee joint; (3) erythrocyte sedimentation rate below <40 mm/h, negative rheumatoid factor; (4) signed informed consent form for participation in the study. Excluded were patients with: (1) presence of crystalline arthropathy; (2) presence of decompensated cardiovascular, pulmonary, renal, or hematological diseases; (3) presence of immunological phenomena.

The study was conducted in adherence to the World Medical Association Declaration of Helsinki (1964) and its revised version (Edinburgh, 2000). All work, including patient data analysis, blood collection and aspiration of synovial fluid, and the content of the informed consent form, was approved by the Committee for Scientific Ethics at the Medical University of Plovdiv, Protocol No 4/10.06.2021.

Arthrocentesis was performed by a rheumatologist on the 156 patients with PsA with hydrops (145 on the knee joint, 6 on the ankle joint, 3 on the lactic joint, and 2 on the shoulder joint) in compliance with antiseptic rules. The synovial fluid obtained via arthrocentesis was examined by two independent rheumatologists using the polarization microscope Leica DM4500P (Leica Mycrosystems Germany, Wetzlar, Germany).

The patients’ disease state was assessed using the Visual Analog Scale for pain intensity (VAS), the Disease Activity for Psoriatic Arthritis (DAPSA), the Psoriatic Arthritis Disease Activity Index (PASDAI), the Composite Psoriatic Disease Activity Index (m CPDAI), and the Health Assessment Questionnaire Disability Index (HAQ-DI). Based on the VAS pain score, the patients were categorized into three groups: patients with moderate pain (40–60 mm<), patients with severe pain (60–80 mm), and patients with very severe pain (>80–100 mm). According to DAPSA, the following categorization of disease activity was applied: low, ≤14; moderate, >14 to ≤28; and high, >28. For PASDAI, disease activity categories were: remission, <1.9; low, >1.9 to 3.2; moderate, >3.2 to 5.4; and high, >5.4. On the basis of mCPDAI, disease activity was categorized as: low, 1 to 3; moderate, >3 to 9; and high, > 9. The HAQ-DI disease activity ranges were: 0 to 1, mild to moderate disability; >1 to 2, moderate to severe disability; >2 to 3, severe to very severe disability.

### Statistical Analysis

Statistical analysis was performed using SPSS version 26.0 (SPSS Inc., Chicago, IL, USA). Continuously measured variables were tested for normality through the Shapiro–Wilk’s test. The normally distributed data were described with means and standard deviations (SD), and comparisons were performed through an independent-samples t-test. Non-normally distributed variables were presented as medians and interquartile ranges (IQR), and analyzed through the Mann–Whitney U test. Categorical and ordinal data were presented as frequencies and percentages, and the Chi-square test was used to establish associations. Odds ratios (OR) and 95% confidence intervals were calculated to show the relation between the presence of crystals and an increased chance for high disease activity. All tests were two-tailed and the results were interpreted as significant at Type I error alpha = 0.05 (*p* < 0.05).

## 3. Results

### 3.1. Comparison of PsA Patients with the Control Group of GoA Patients

The PsA patients had a significantly higher BMI and a higher prevalence of diabetes, ischemic heart disease, hyperuricemia, dyslipidemia, and obesity in comparison to the GoA patients. The presence of crystals in the synovial fluid was significantly associated with PsA when compared to the GoA group, where no crystals were detected, *p* < 0.001. Crystals of monosodium urate (MSU) were found in 25 (67.58%) patients with PsA; pyrophosphate crystals (CPP) were found in eight (21.62%) patients with PsA; lipid drops were found in two (5.4%) patients with PsA. Two (5.4%) patients with PsA had both MSU and CPP crystals (Table 1).

### 3.2. Comparison of PsA Patients with and without Crystals in the Synovial Fluid

The PsA patients with and without crystals had similar age and sex distribution. Ischemic heart disease and hyperuricemia were significantly more frequent in the PsA patients with crystals (*p* < 0.001 for both conditions). No significant differences existed in relation to hypertension (*p* = 0.209), diabetes (*p* = 1.00), dyslipidemia (*p* = 0.849), obesity (*p* = 0.084), smoking (*p* = 0.586), CУE (*p* = 0.145), and CRP (*p* = 0.258).

Diagnosis duration was not significantly associated with the presence or absence of crystals (*p* = 0.614). No significant differences were observed in relation to polyarticular (*p* = 0.848) and oligoarticular involvement (*p* = 0.453), skin psoriasis (*p* = 0.245), and enthesitis (*p* = 0.493). Nail involvement and dactylitis were significantly more frequent in the PsA patients with crystals (*p* < 0.001 for both comparisons) (Table 2).

### 3.3. Relationship between the Presence of Crystals in the Synovial Fluid of PsA Patients and Disease Activity Indices

All disease activity indices showed significant associations with the presence or absence of crystals in the synovial fluid of the PsA patients (Table 3). According to PtVAS, the majority of the PsA patients with crystals had severe pain, whereas the majority of the PsA patients without crystals had mild to moderate pain, *p* < 0.001.

Based on DAPSA, 75.70% of the PsA patients with crystals had high disease activity versus 16% of the PsA patients without crystals, *p* < 0.001. PASDAI showed a similar distribution of the PsA patients with crystals among low, moderate and high disease activity, whereas the PsA patients without crystals were mostly associated with low disease activity, *p* < 0.001. According to mCPDAI, 67.60% of the PsA patients with crystals had high disease activity, while 75.60% of the PsA patients without crystals had low disease activity, *p* < 0.001. HAQ-DI showed severe disability in 59.50% of the PsA patients with crystals versus 9.20% of the PsA patients without crystals, *p* < 0.001.

**Figure 1:**
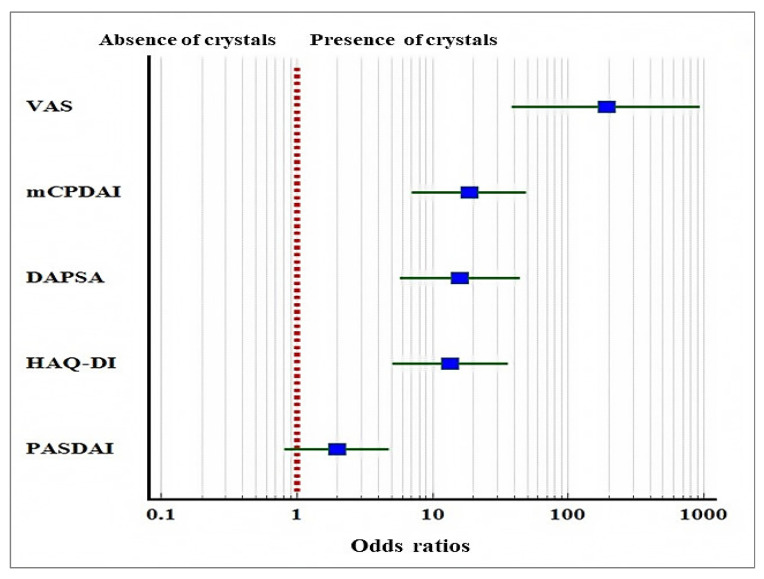
Odds ratios and 95% CIs showing the relation between the presence of crystals in the synovial fluid of the PsA patients and the chance of severe and unbearable pain (VAS), high disease activity (mCPDAI and DAPSA), and severe disability (HAQ).

We estimated the odds ratios between the presence of crystals and the incidence of high disease activity and severe pain (Figure 1). The presence of crystals was associated with an increased chance for severe pain (OR = 157.25, 95% CI: 39.50 to 625.94, *p* < 0.001); high disease activity on mCPDAI (OR = 18.75, 95% CI: 7.13 to 49.25, *p* < 0.001) and DAPSA (OR = 15.96, 95% CI: 5.76 to 44.23, *p* < 0.001); severe disability on HAQ-DI (OR = 13.60, 95% CI: 5.09 to 36.31, *p* < 0.001). On the PASDAI scale, the presence of crystals was not significantly associated with an increased chance for high disease activity (OR = 1.97, 95% CI: 0.81 to 4.79, *p* = 0.133).

## 4. Discussion

Our first goal was to compare the incidence of synovial fluid crystals in patients with PsA to a control group of patients with GoA. The results showed a strong association between hyperuricemia and the presence of crystals in the synovial fluid of PsA patients. The high rate of hyperuricemia (71.15%) in the PsA group is consistent with previous studies that found elevated uric acid levels in the serum of PsA patients [5,6,7].

Given the scarcity of scientific literature on the characteristics of PsA patients’ synovial fluid [9,10], the results of polarization microscopy on 156 patients with PsA and joint hydrops shed light on an understudied issue. Our data showed a higher rate of synovial crystals in comparison to Oliviero et al. [15], who found MSU crystals in 3.34% of PsA patients, and also in comparison to Galozzi et al., who observed crystals in 9.53% of PsA patients [16]. The different rates of synovial fluid crystals in the research literature are difficult to explain and more research is needed to validate these findings.

Moreover, we observed a very strong association between the presence of crystals and disease activity indices in the PsA patients, as well as an increased chance for severe pain. The odds ratio for VAS pain yielded a high value, which we explain by the almost perfect fit of the data, where 90% of the patients with crystals experienced severe pain versus a very small proportion of the patients with crystals. Two disease activity indices, mCPDAI and DAPSA, were consistent in showing a strong link between the presence of crystals in PsA patients’ synovial fluid and high disease activity.

Our findings differ from previous studies that found a weak or non-existent association between hyperuricemia and disease activity in PsA patients [17,18]. Aside from variations due to patients’ ethnicity, demographic, and lifestyle characteristics [17], another plausible explanation for the difference is that we tested the relationship between synovial fluid crystals and disease activity indices, whereas the other studies tested serum uric acid levels.

Hyperuricemia is a common comorbidity associated with PsA that the majority of patients with this diagnosis are likely to have, as was the case in our study. This may have reduced the variability within the studied sample, which is described as one of the reasons for low correlation coefficients in the statistical literature [19].

Current research has not been able to fully explain the underlying factors for the presence of crystals in the synovial fluid of PsA patients, nor to draw a clear distinction between PsA and gout [5]. The two disease conditions are closely linked by shared pathophysiology, common clinical symptoms, and radiological findings that present a challenge for daily clinical practice due to their confounding influences [20].

While urate-lowering therapy is commonly used to treat gout, its appropriate use and efficacy in PsA patients is dependent on an accurate diagnosis of the patient’s condition.

Our findings show that synovial fluid crystals have a strong negative impact on the health-related quality of life of PsA patients due to an increased risk of severe pain, high disease activity, and severe disability.

On this basis, we believe that synovial fluid examination should be included in the diagnostic and monitoring procedures for PsA patients with active disease, to aid in determining whether urate-lowering therapy is required.

## 5. Conclusions

Our findings suggest that the presence of crystals in synovial fluid could be a sign of increased disease activity and deteriorating physical ability in PsA patients. The examination of synovial fluid can help clinicians treat and monitor patients with active disease.

## Figures and Tables

**Table 1:** Demographic and clinical data about the PsA and GoA patients.

Variables	Groups		*p*
	PsA Patients	GoA Patients	
	(*n* = 156)	(*n* = 50)	
Age (years)			
Mean ± SD	54.36 ± 13.55	62.46 ± 11.55	<0.001 ^t^
Sex *n* (%)			
Women	63 (40.40%)	25 (50%)	
Men	93 (59.60%)	25 (50%)	0.253 ^f^
BMI	31.40 ± 5.44	28.36 ± 4.33	<0.001 ^t^
Comorbidity *n* (%)			
Hypertension	112 (71.70%)	41(82.00%)	0.193 ^f^
Diabetes	125 (80.12%)	13 (26.00%)	<0.001 ^f^
Ischemic h.d.	61 (39.10%)	9 (18.00%)	0.006 ^f^
Hyperuricemia	111 (71.15%)	3 (6.0%)	<0.001 ^f^
Dyslipidemia	99 (63.46%)	13 (26.00%)	<0.001 ^f^
Obesity	132 (84.60%)	25 (50.00%)	<0.001 ^f^
Synovial fluid crystals *n* (%)	37 (23.70%)	0 (0.00%)	<0.001 ^f^

^t^—independent samples t-test; ^f^—Fisher’s exact test.

**Table 2:** Demographic, clinical, and imaging data about the PsA patients with and without crystals.

Variables	Groups		*p*
	PsA with	PsA without	
	Crystals	Crystals	
	(*n* = 37)	(*n* = 119)	
Age (years)			
Mean ±S D	55.02 ± 14.78	54.16 ± 13.20	0.735 ^t^
Sex *n* (%)			
Women	12 (32.43%)	51(42.85%)	
Men	25(67.56%)	68 (57.15%)	0.261 ^f^
BMI	29.91 ± 5.31	31.88 ± 5.42	0.056 ^t^
Comorbidity			
Hypertension	30 (81.10%)	82 (68.90%)	0.209 ^f^
Diabetes	30 (81.10%)	95 (79.80%)	1.000 ^f^
Ischemic h.d.	26 (70.30%)	35 (29.41%)	<0.001 ^f^
Hyperuricemia	37 (100.00%)	74 (62.18%)	<0.001 ^f^
Dyslipidemia	23 (62.20%)	76 (63.90%)	0.849 ^f^
Obesity	30 (91.89%)	102 (85.70%)	1.000 ^f^
Smoking *n* (%)	13 (35.10%)	52(43.70%)	0.586 ^f^
CУE mm/h			
Median (IQR)	45.00 (35.00)	35.00 (35.00)	0.146 ^U^
CRP g/l			
Median (IQR)	51.00 (20.00)	45.00 (35.00)	0.101 ^U^
Diagnosis duration			
Mean ± SD	11.23 ± 7.9	10.55 ± 3.5	0.614 ^t^
Involvement *n* (%)			
Polyarticular	14 (37.83%)	43 (36.10%)	0.848 ^f^
Oligoarticular	19 (51.35%)	52 (43.80%)	0.453 ^f^
Skin psoriasis *n* (%)	35 (94.59%)	103 (86.55%)	0.245 ^f^
Nail involvement *n* (%)	33 (89.18%)	66 (66.66%)	<0.001 ^f^
Dactylitis *n* (%)	35 (94.59%)	13 (10.92%)	<0.001 ^f^
Enthesitis *n* (%)	31 (83.78%)	92 (77.31%)	0.493 ^f^

^t^—independent samples t-test; ^f^—Fisher’s exact test; ^U^—Mann–Whitney U test.

**Table 3:** PsA patients with and without crystals across stages of disease activity.

Disease Activity Indices	PsA with	PsA without	*p*
	Crystals (*n* = 37)	Crystals (*n* = 119)	
VAS pain *n* (%)			
mild	2 (5.40%)	90 (75.60%)	
moderate	1 (2.70%)	21 (17.60%)	
severe	15 (40.50%)	6 (5.00%)	
unbearable	19 (51.40%)	2 (1.70%)	<0.001
DAPSA *n* (%)			
low disease activity	6 (16.20%)	65 (54.60%)	
moderate disease activity	3 (8.10%)	35 (29.40%)	
high disease activity	28 (75.70%)	19 (16.00%)	<0.001
PASDAI *n* (%)			
low disease activity	12 (32.40%)	77 (64.70%)	
moderate disease activity	12 (32.40%)	39 (32.80%)	
high disease activity	13 (35.10%)	3 (2.50%)	<0.001
mCPDAI *n* (%)			
low disease activity	8 (21.60%)	90 (75.60%)	
moderate disease activity	4 (10.80%)	14 (11.80%)	
high disease activity	25 (67.60%)	15 (12.60%)	<0.001
HAQ-DI *n* (%)			
mild disability	10 (27.00%)	68 (57.10%)	
moderate disability	5 (13.50%)	40 (33.60%)	
severe disability	22 (59.50%)	11 (9.20%)	<0.001

## Data Availability

The data presented in this study are available on request from the corresponding author. The data are not publicly available due to institutional restrictions.

## References

[1] Alamanos Y., Voulgari P.V., Drosos A.A. (2008). Incidence and prevalence of psoriatic arthritis: A systematic review. J. Rheumatol..

[2] Augustin M., Vietri J., Tian H., Gilloteau I. (2017). Incremental burden of cardiovascular comorbidity and psoriatic arthritis among adults with moderate-to-severe psoriasis in five European countries. J. Eur. Acad. Dermatol. Venereol..

[3] Arevalo A.B., Haddadin F., Contreras G., Murray S., Luo Y., Ali Y. (2019). Cardiovascular impact of hyperuricemia in patients with psoriatic arthritis. Ann. Rheum. Dis..

[4] Perez-Chada L.M., Merola J.F. (2020). Comorbidities associated with psoriatic arthritis: Review and update. Clin. Immunol..

[5] Tripolino C., Ciaffi J., Ruscitti P., Giacomelli R., Meliconi R., Ursini F. (2021). Hyperuricemia in Psoriatic Arthritis: Epidemiology, Pathophysiology, and Clinical Implications. Front. Med..

[6] Kamiya K., Oiso N., Kawada A., Ohtsuki M. (2021). Epidemiological survey of the psoriasis patients in the Japanese Society for Psoriasis Research from 2013 to 2018. J. Dermatol..

[7] Isha Jain V.K., Lal H. (2011). C-reactive protein and uric acid levels in patients with psoriasis. Indian J. Clin. Biochem..

[8] Tsuruta N., Imafuku S., Narisawa Y. (2017). Hyperuricemia is an independent risk factor for psoriatic arthritis in psoriatic patients. J. Dermatol..

[9] Swan A., Amer H., Dieppe P. (2002). The value of synovial fluid assays in the diagnosis of joint disease: A literature survey. Ann. Rheum. Dis..

[10] Boumans D., Hettema M.E., Vonkeman H.E., Maatman R.G., van de Laar M.A. (2017). The added value of synovial fluid centrifugation for monosodium urate and calcium pyrophosphate crystal detection. Clin. Rheumatol..

[11] Hollander J.L., Reginato A., Torralba T.P. (2001). Examination of synovial fluid as a diagnostic aid in arthritis. Classic Papers in Rheumatology.

[12] Lai Y.C., Yew Y.W. (2016). Psoriasis and uric acid: A population-based cross-sectional study. Clin. Exp. Dermatol..

[13] Kwon H.H., Kwon I.H., Choi J.W., Youn J.I. (2011). Cross-sectional study on the correlation of serum uric acid with disease severity in Korean patients with psoriasis. Clin. Exp. Dermatol..

[14] Solak B., Dikicier B.S., Erdem T. (2017). Impact of elevated serum uric acid levels on systemic inflammation in patients with psoriasis. Angiology.

[15] Oliviero F., Scanu A., Galozzi P., Gava A., Frallonardo P., Ramonda R., Punzi L. (2013). Prevalence of calcium pyrophosphate and monosodium urate crystals in synovial fluid of patients with previously diagnosed joint diseases. Joint Bone Spine.

[16] Galozzi P., Oliviero F., Frallonardo P., Favero M., Hoxha A., Scanu A., Lorenzin M., Ortolan A., Punzi L., Ramonda R. (2016). The prevalence of monosodium urate and calcium pyrophosphate crystals in synovial fluid from wrist and finger joints. Rheumatol. Int..

[17] Lai T.L., Yim C.W., Wong P.Y., Leung M.C., Ng W.L. (2018). Hyperuricemia in Asian psoriatic arthritis patients. Int. J. Rheum. Dis..

[18] Bruce I.N., Schentag C.T., Gladman D.D. (2000). Hyperuricemia in psoriatic arthritis: Prevalence and associated features. J. Clin. Rheumatol..

[19] Goodwin L.D., Leech N.L. (2006). Understanding Correlation: Factors that Affect the Size of r. J. Exper. Educ..

[20] Felten R., Duret P.M., Gottenberg J.E., Spielmann L., Messer L. (2020). At the crossroads of gout and psoriatic arthritis: “psout”. Clin. Rheumatol..

